# Abnormal Uterine Bleeding Among Rural Adolescent Schoolgirls: A Cross-Sectional Study

**DOI:** 10.3390/medicina61010033

**Published:** 2024-12-28

**Authors:** Yasir Salih, Ghaday S. Almutairi, Norah H. Alhumaidi, Nadiah Alhabardi, Ishag Adam

**Affiliations:** 1 Faculty of Medicine, University of Khartoum, P.O. Box 102, Khartoum 11111, Sudan; dryasirsalih@yahoo.ie; 2 College of Medicine, Qassim University, Buraydah 52571, Saudi Arabia; ghadialoqaili@gmail.com (G.S.A.); noura.h.alhumaidi.19@gmail.com (N.H.A.); 3 Department of Obstetrics and Gynecology, College of Medicine, Qassim University, Buraydah 52571, Saudi Arabia; ia.ahmed@qu.edu.sa

**Keywords:** adolescent, age, menstrual cycle patterns, menstrual irregularities, abnormal uterine bleeding

## Abstract

*Background and Objectives*: The International Federation of Gynecology and Obstetrics (FIGO) and the American College of Obstetricians and Gynecologists (ACOG) define abnormal uterine bleeding (AUB) as “bleeding from the uterus that is abnormal in regularity, volume, frequency, or duration and occurs in the absence of pregnancy”. The impact of AUB on the physical and psychosocial well-being of adolescent girls can be significant. In this study, we aim to investigate the menstrual cycle characteristics in adolescent Sudanese schoolgirls and the prevalence of abnormal uterine bleeding (AUB) and its associated factors. *Materials and Methods*: A school-based cross-sectional study was conducted in Almatamah locality, Sudan. A questionnaire was used to collect sociodemographic data and menstrual cycle parameters. Weight and height were measured, body mass index (BMI) was calculated, and hemoglobin levels were determined. Logistic regression was also performed. *Results*: Of the 162 girls in the study, 27.2% had frequent cycles, 3.7% had infrequent cycles, 2.5% had prolonged menses duration, 44.4% had irregular cycles, and 21.0% had heavy menstrual bleeding. The overall prevalence of AUB in the study was 64.8%. None of the investigated factors (chronological age, maternal education or occupation, gynecological age, BMI, and hemoglobin level) were associated with AUB. *Conclusions*: In conclusion, the prevalence of AUB was high among the study participants, with irregular cycles, frequent cycles, and heavy menstrual bleeding being the most common types.

## 1. Introduction

The International Federation of Gynecology and Obstetrics (FIGO) and the American College of Obstetricians and Gynecologists (ACOG) define abnormal uterine bleeding (AUB) as “bleeding from the uterus that is abnormal in regularity, volume, frequency, or duration and occurs in the absence of pregnancy” [1,2]. The condition is commonly referred to in the literature as irregular cycles or menstrual irregularities [3,4,5]. AUB is a health problem often observed in adolescent girls, with varied differential diagnoses, e.g., pregnancy, infections, anovulation [6]. In adolescent girls, the cause is mostly immaturity or dysregulation of the hypothalamic-pituitary-ovarian axis. It is regarded as normal physiology in this age group [2,7]. Evaluation of AUB in adolescent girls is necessary to detect common etiologies, such as non-structural causes, which are different from etiologies more common during the later ages of adult women and those of reproductive age [6]. AUB in adolescent girls can appear early and serve the first sign of undetected bleeding disorders [8]. Therefore, a detailed medical history and physical and gynecological examination are necessary to assess AUB in adolescent girls [9].

The impact of AUB on the physical and psychosocial well-being of adolescent girls can be significant. Girls with AUB may experience fatigue and reduced academic performance [4,10]. Furthermore, AUB can negatively affect their social and emotional well-being, leading to decreased self-esteem and social isolation [11]. According to the United Nations Educational, Scientific, and Cultural Organization (UNESCO), 10% of girls in Africa miss school due to inadequate access to menstrual products or a lack of suitable restroom facilities at school [12]. AUB can also indicate underlying health conditions, such as polycystic ovary syndrome or thyroid dysfunction, which can have long-term health consequences if left untreated [13]. Therefore, proper assessment and management of AUB in adolescent girls is highly needed [14].

Several studies have investigated the worldwide prevalence of AUB. It has been reported that almost 50% of all women have had AUB at some point in their lives, and the condition accounts for half of the gynecologic problems observed among adolescents [13,15]. Depending on the age group of the population studied, different authors have reported a different prevalence of AUB. For example, studies in Ethiopia have reported a prevalence between 30% and 50% [3,4,5,16]. In Egypt, the reported prevalence is between 40% [17] and 95% [18]. Similar findings have also been reported in studies conducted in Asia [19,20,21], Europe [22], and South America [23]. Several factors, such as chronological age, gynecological age, body mass index (BMI), and hemoglobin levels, have been reported to be associated with AUB in adolescents [3,5,17,20,22].

Despite this growing recognition of AUB’s impact on adolescents in developing and well-developed countries, studies on this topic in Sudan are limited. Menstrual health is likely a significant issue among adolescent girls in this country despite the limited data [24]. Studies about the situation are highly needed to guide those involved in adolescents’ health care, such as school nurses, doctors, and educators. This study aimed to investigate the prevalence of AUB in adolescent girls in Sudan and the associated factors.

## 2. Materials and Methods

### 2.1. Sampling and Recruitment

**Figure 1:**
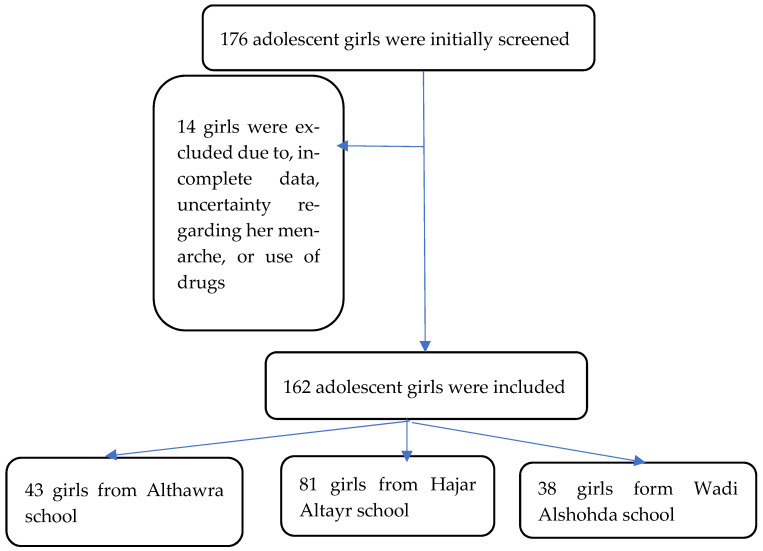
Flow chart diagram of the study.

The approval to run this study in schools was obtained from the local education authority after presenting the study’s ethical approval. Then, permission was obtained from each school manager. We enrolled a sample of adolescent girls conveniently selected from the three schools located in the Almatamah locality (130 km from Khartoum, the capital of Sudan), a rural area in the northern region of Sudan, between May and September 2022. The schools were Hajar Altayr, Wadi Alshohda, and Althawra, and were located in Wad Hamid district, one of the three districts in the Almatamah locality (Figure 1).

### 2.2. Inclusion Criteria

All girls who had attained menarche were enrolled in the targeted schools, provided written consent from a parent or guardian, and were eligible to participate in the study.

### 2.3. Exclusion Criteria

Premenarchal girls, those with a significant or chronic medical condition or bleeding disorder, those who were married or pregnant or taking hormonal medication, and those who did not provide written consent were excluded from the study.

This study employed a cross-sectional design. The girls were approached in their classrooms and informed about the aim of the study. Before the questionnaire distribution, a consent form was distributed to those eligible for the research and wishing to participate, to be signed by their parents. Then, those who returned the consent were invited to complete the study questionnaire and measure their weight, height, and hemoglobin level. If a parent had any questions regarding the study or the study procedure, they were answered physically, and the research team cleared their doubts. Two trained female medical officers supplied the questionnaire through face-to-face interviews and measured the participants’ weight and height in privacy in one of the school offices without disrupting classes. Three visits were needed for Hajar Altayr, and two for Wadi Alshohda and Althawra. Then, 3 mL of blood was obtained from each participant, collected into a vial, and sent to the laboratory to measure hemoglobin levels, coded, and recorded on a form designed for physiological parameters. Weight was measured after removing shoes and heavy clothing or objects using a standard medical weighing scale. Height was measured by a stadiometer with bare feet and the head in the Frankfurt horizontal plane.

The investigators designed a questionnaire to collect data for this study. The questionnaire was pre-tested on ten volunteer students in one school similar to the target population. The results were used to rephrase and modify some parts of the questionnaire, making it clearer to the participants. The questionnaire consisted of sociodemographic information, including age in years and maternal education (<secondary or ≥secondary) level. Subsequently, participants were asked about menstrual characteristics, age at menarche, and menstrual irregularities. Weight and height were measured using established procedures, and BMI was computed using the conventional formula of weight in kilograms divided by the square of height in meters. Hemoglobin levels were also measured.

Girls found to have menstrual disorders requiring medical attention were referred to the health center in Almatamah.

### 2.4. Definitions

The revised 2018 FIGO system for the nomenclature of symptoms of normal uterine bleeding and AUB in the reproductive years (FIGO AUB System 1) was used [1]. The study defines cycle frequency as infrequent (>38 days), average (24–38 days), and frequent (<24 days). The duration is described as normal (≤8 days) or prolonged (>8 days). Regularity is categorized as normal or “regular” (a shortest to longest cycle variation of ≤7–9 days) or irregular (a shortest to longest cycle variation of 10–28 days). However, while the FIGO definition of regularity states a variation of 7–9 days, depending on age (18–25 years ≤9 days; 26–41 years ≤7 days; 42–45 years ≤9 days), there is no mention of those less than 18 years of age; thus, in this study, we considered any variation of less than nine days as regular. The participants self-reported the flow volume as light, normal, or heavy (excessive menstrual blood loss that negatively impacts a woman’s physical, social, emotional, and material quality of life).

### 2.5. Sample Size

A sample size of 162 adolescent girls was calculated based on the assumption of the prevalence (15.0%) of AUB among adolescent girls, which had been reported in the neighboring country (Egypt). This sample size was calculated using a single proportional formula, (n = Z2pq/d2), where q = (1 − p), Z1 − α = confidence interval of 95% = 1.96, and d = 5%, margin of error = 0.05.

### 2.6. Statistical Analysis

Data were analyzed using the Statistical Package for the Social Sciences (IBM SPSS, version 25.0). The normality of distribution for continuous data was tested using a Shapiro–Wilk test, which was found to be normally distributed and expressed as mean and standard deviation (SD). Descriptive statistics were obtained for demographic variables, menstrual characteristics, and all measurements. Univariate logistic regression analyses were utilized to assess the association between various independent variables, such as age, hemoglobin level, BMI, gynecological age, mother’s occupation, mother’s education, and the presence or absence of AUB. The odds ratios (OR) and their corresponding 95% confidence intervals (95% CI) were calculated. A *p*-value of less than 0.05 was considered statistically significant.

## 3. Results

### 3.1. Demographic and Social Characteristics of Participants

A total of 162 adolescent girls participated in the study. Their ages ranged between 12.78 and 18.87 years old, with a mean (SD) age of 15.78 (1.25). The mean (SD) gynecological age was 2.28 (1.37) years old, and the mean (SD) BMI was 19.90 (3.36) kg/m^2^. The mean (SD) hemoglobin level was 12.58 (1.25) g/dL, and the mean (SD) menstrual duration was 5.23 (1.38) days. Ninety percent of mothers were homemakers, 26.5% were illiterate, and 61.7% had a fundamental educational level; see Table 1.

### 3.2. Menstrual Patterns and Disturbances

Of the 162 girls, 27.2% reported frequent cycles, 3.7% reported infrequent cycles, 2.5% reported prolonged menses duration, 44.4% reported irregular cycles, and 21.0% reported heavy menstrual bleeding (HMB); see Table 2.

### 3.3. Factors Associated with AUB

The overall prevalence of AUB in the study was 64.8%. Univariate analysis did not reveal significant associations between AUB and chronological age, maternal education or occupation, gynecological age, BMI, or hemoglobin level; see Table 3.

## 4. Discussion

The study looked at the four basic characteristics of the menstrual cycle (i.e., frequency, menses duration, regularity, and flow volume) and classified them according to the FIGO AUB System 1, 2018 revision. We found an overall prevalence of 64.8% for AUB among the girls in this study. Our findings are similar to what Hossam et al. reported in secondary school girls in Egypt (42%–65% prevalence) [17]. Our findings are also consistent with findings from studies outside the region, such as the studies by Abbasi et al. from Pakistan (69% prevalence) [19] and Sharma et al. from Nepal (64.2% prevalence) [20]. The prevalence in our study was higher than that in several studies from Ethiopia that reported a prevalence between 32% and 46% [3,5,16]. It is worth mentioning here that these Ethiopian studies focused on college or university students, an age group older than our study. The most common cause of AUB in adolescents is the immaturity or dysregulation of the hypothalamic–pituitary–ovarian axis, which may explain the decrease in prevalence with increasing age of the sample populations in the studies mentioned above.

We found that 69.1% of the girls had an average cycle frequency of 24–38 days, comparable to Zeru et al. [5] and Pitangui et al. [23]’s findings in Ethiopia and Brazil. The prevalence of frequently occurring cycles (<24 days) in this study was 27.2%, which is also consistent with what the previous two authors reported, but is higher than what was reported by Abdelmoty et al. in Egypt (15.0% prevalence) [18], by Ghandour et al. among Palestinian adolescent refugees in Jordan (2.6% prevalence) [25], and by other authors from Ethiopia (2.0%–5.0% prevalence) [3,4,5,16]. However, this study found infrequent cycles in 3.7% of adolescent girls, consistent with the Ethiopian findings but lower than what Abdelmoty et al. found in Egyptian adolescents (27.8% prevalence). The importance of this finding is that more frequent cycles expose adolescent girls to an increased risk of anemia, as Zeru et al. found [5]. Furthermore, infrequent cycles are associated with ovulatory conditions such as polycystic ovarian syndrome (PCOS), a spectrum that requires a different approach in terms of health services planning.

The majority (97.5%) of our participants had a normal menses duration of 8 days or less, with a mean (SD) of 5.23 (1.38) days. Elshiekh et al. found a mean (SD) duration of 4.8 (1.9) days among adolescent school girls in north Sudan [24]. Arafa et al. reported a mean (SD) in terms of the menses of 5.2 (1.4) days among adolescent school girls in Egypt [26]. Other authors have reported similar percentages in southwestern Nigeria [27], in northwestern Ethiopia [28], and in Egypt [18]. The prevalence of prolonged periods in this study was 2.5%, which is comparable to the other studies in the region [3,5,25,29]

In this study, 44.4% of girls had irregular cycles (a shortest to longest cycle variation of 10–28 days), which made this the most common type of AUB. Adam et al. reported comparable findings [29] among nursing students in Sudan (55% prevalence) and by Zegeye et al. [28] in Ethiopia (42% prevalence). Zeru et al. [5] reported a much lower prevalence of 19.0–20.0% among college and university students in Debre Berhan, Ethiopia, but the age of their participants (mean 20.6 years) was higher than our study (mean 15.78 years), which may explain the noticeable difference in prevalence.

The flow volume in this study was heavy in 21.0% of the participants. The reported prevalence of HMB varies widely in the literature. Demeke et al. in Ethiopia [4], Abdelmotyet al. in Egypt [18], and Pitangui et al. in Brazil [23] reported a prevalence of 19.8%, 17.29, and 21.9%, respectively, consistent with our findings. Other authors, such as Amu et al. in Nigeria, reported a much higher prevalence of 57.4%. Furthermore, a prevalence of 4.0% to 12.0% was reported by Zeru et al. [5] and Shiferaw et al. in Ethiopia [16], and Ghandour et al. in Jordan [25]; these rates are lower than our findings. We assume that the method used to define HMB significantly contributed to these discrepancies [1].

Our study found no significant associations between AUB and chronological age, gynecological age, maternal occupation or education, BMI, or hemoglobin level. Similarly, Zeru et al. in Ethiopia and Sharma et al. in Nepal did not find associations between chronological age and AUB, while Agarwal et al. in Singapore did not find associations with gynecological age in their study populations, which is in agreement with our findings [5,20]. Marques et al. in Portugal found no association between AUB and BMI in adolescent girls [22].

However, numerous authors have found significant associations between AUB and these factors. For example, Mittiku et al., Agarwal et al., and Marques et al. found an increase in the occurrence of AUB with a decrease in chronological age and gynecological age [3,21,22]. Additionally, Agarwal et al., Mittiku et al., and Hossam et al. reported an association between increased AUB and BMI [3,17,21]. In Ethiopia, Zeru et al. found an increased prevalence of AUB among university students with higher BMI and low hemoglobin levels [5]. Anemia was reported to increase the odds of menstrual irregularity in Bangladesh (AOR = 2.1) [30]. Several factors could explain the association between anemia and AUB [31,32]. However, the direction of the association between AUB and anemia has yet to be settled [31,32]. Longitudinal studies are needed to explore the direction between anemia and AUB.

In the current study, maternal education was not associated with AUB. Previous studies have shown that education is an essential part of a girl’s attitude toward menstrual and puberty health [33,34]. We did not observe significant associations between AUB and chronological age, maternal education or profession, gynecological age, BMI, or hemoglobin level. Sociodemographic and genetic differences could explain the difference between our study’s results and those of the other studies in the populations.

### Strengths and Limitations of This Study

The findings of this study are important to guide researchers, educators, planners, nurse practitioners, and other health professionals about AUB in adolescent Sudanese girls. However, there are some limitations. The study was conducted in one rural locality in north Sudan, which has little diversity in ethnicity and socio-economic status, which may not correctly represent the whole country. The study population did not include adolescents who were married but may have AUB or other menstrual irregularity, and also excluded girls who were taking hormonal medication (COC may be prescribed in cases of AUB). The questionnaire that was used in the current study needed to be validated. Furthermore, the reliance on participants’ recall of events and the sample size made analyzing and interpreting many associated factors difficult, thus necessitating further larger studies on the topic.

## 5. Conclusions

The menstrual cycle pattern of adolescent Sudanese girls observed in this study is consistent with worldwide patterns. The prevalence of AUB was high among the study participants, with irregular cycles, frequent cycles, and HMB being the most common types. These findings highlight the need for increased attention to adolescent menstrual health in Sudan and for further large-scale studies to better understand this issue.

## Figures and Tables

**Table 1:** Socio-demographic characteristics of adolescent girls (*n* = 162), Sudan, 2022.

Variable	Mean (Standard Deviation)
Age, years	15.78 (1.25)
Body mass index, kg/m^2^	19.90 (3.36)
Gynecological age, years	2.28 (1.37)
Hemoglobin level, g/dL	12.58 (1.25)
Manses Duration, days	5.23 (1.38)
	Frequency (proportion)
Mother’s educational level	
Illiterate	43 (26.5)
Basic	100 (61.7)
Secondary or Higher	19 (11.7)
Mother’s occupation	
Housewife	146 (90.1)
Employee	16 (9.9)

**Table 2:** Prevalence of abnormal uterine bleeding (AUB) in adolescent girls (*n* = 162), Sudan, 2022.

Variable	Frequency	Proportion	95% Confidence Interval
Cycle frequency			
Normal (24–38 days)	112	69.1	61.7–75.7
Frequent (<24 days)	44	27.2	20.9–34.5
Infrequent (>38 days)	6	3.7	1.7–7.8
Menses duration			
Normal (≤8 days)	158	97.5	93.8–99.0
Prolonged (>8 days)	4	2.5	1.0–6.2
Cycle regularity			
Irregular (variation of 28–10 days)	72	44.4	37.0–52.1
Regular (variation of ≤7–9 days)	90	55.6	47.9–63.0
Menstrual flow volume			
Heavy	34	21.0	15.4–27.9
Normal	128	79.0	72.1–84.6
Intermenstrual bleeding			
Yes	20	12.3	08.1–18.3
No	142	87.7	81.7–91.9
Overall AUB (Presence of at least one abnormal finding)			
No AUB	57	35.2	28.3–42.8
Yes AUB	105	64.8	57.2–71.7

**Table 3:** Univariate analysis of factors associated with abnormal uterine bleeding (AUB) in adolescent girls (*n* = 162), Sudan, 2022.

Variable		Adolescents with No AUB (*n* = 57)	Adolescents with AUB (*n* = 105)		
		**Mean (Standard Deviation)**		**OR (95% CI)**	***p***
Hemoglobin level, g/dL		12.63 (1.15)	12.55 (1.30)	0.94 (0.70–1.25)	0.682
Age, years		15.61 (1.26)	15.88 (1.25)	1.06 (0.76–1.48)	0.71
Body mass index, kg/m^2^		19.57 (3.15)	20.08 (3.46)	1.04 (0.93–1.15)	0.434
Gynecological age, years		2.04 (1.18)	2.41 (1.45)	1.21 (0.87–1.67)	0.243
		**Frequency (percentage)**			
Mother’s occupation	Housewife	51 (89.5)	95 (90.5)	Reference	
	Employee	6 (10.5)	10 (9.5)	0.73 (0.21–2.46)	0.616
Mother’s education level	Illiterate or basic	52 (91.2)	91 (86.7)	Reference	
	Secondary or more	5 (8.8)	14 (13.3)	1.73 (0.50–5.91)	0.381

Abbreviations: SD = standard deviation; OR = odds ratio; 95% CI = 95% confidence interval.

## Data Availability

Data will be available from the correspondence author upon reasonable request.
